# Secular Changes in Cranial Morphology and Pattern of Sexual Dimorphism in Modern Japanese: A Geometric Morphometric Analysis Using Post‐Mortem Computed Tomography Data

**DOI:** 10.1002/ajpa.70235

**Published:** 2026-04-03

**Authors:** Shiori Usui, Hideki Amano, Hideyuki Hayakawa, Seiji Shiotani, Naomichi Ogihara

**Affiliations:** ^1^ Laboratory of Human Evolutionary Biomechanics, Department of Biological Sciences Graduate School of Science, the University of Tokyo Bunkyo‐ku Tokyo Japan; ^2^ Second Biology Section, First Department of Forensic Science National Research Institute of Police Science Kashiwa‐shi Chiba Japan; ^3^ Department of Forensic Medicine Tsukuba Medical Examiner's Office Tsukuba‐shi Ibaraki Japan; ^4^ Department of Radiology Seirei Fuji Hospital Fuji‐shi Shizuoka Japan

**Keywords:** brachycephalization, cranium, forensic anthropology, sex estimation, skull

## Abstract

**Objectives:**

Although human cranial morphology is thought to have undergone changes in various populations over the past century, the details of this three‐dimensional transformation remain largely unknown. To address this problem, we conducted a geometric morphometric analysis of cranial shapes in the historical Japanese group who lived approximately 100 years ago and the present‐day Japanese group who died in recent years.

**Materials and Methods:**

We used a total of 112 computed tomography (CT) scan data: CT scans of 56 historical Japanese dry crania from individuals who lived in the late 19th to early 20th century and postmortem CT data of 56 present‐day Japanese who died in the 2020s. Each cranium was represented by 161 landmarks, and their coordinates were normalized by centroid size and registered using generalized Procrustes analysis to remove variation due to uniform scaling, translation, and orientation. Principal component analysis was then conducted to explore shape variation among the specimens.

**Results:**

Comparison between these two populations revealed clear secular changes, including brachycephalization and enlargement of the mastoid processes. Furthermore, our results indicate that certain forms of sexual dimorphism, such as larger mastoid processes and more prominent external occipital protuberances in males, have become more pronounced over time.

**Discussion:**

These findings demonstrate that the human cranium has undergone substantial changes over the last century, likely influenced by shifts in environmental, nutritional, and lifestyle factors. They can also contribute to improving the accuracy of sex estimation for unidentified skeletal remains in both forensic and archeological contexts.

## 1 Introduction

It is common in anthropology to use documented human skeletal collections from the late 19th and early 20th centuries as “modern” reference samples. In the United States, the Hamann–Todd collection (Cleveland Museum of Natural History; Mensforth and Latimer 1989) and the Robert J. Terry collection (Smithsonian Institution, National Museum of Natural History; Hunt and Albanese 2005) are widely used because individuals have known age at death, sex, ancestry, and often cause of death (Campanacho et al. 2021). Using these materials, many standard methods were developed and tested, for example, sex estimation from the cranium and pelvis (e.g., Giles and Elliot 1963; Klales et al. 2012; Ubelaker and Volk 2002; Walker 2008) and adult age estimation (e.g., Lovejoy et al. 1985; Meindl and Lovejoy 1985), and they remain common reference samples in forensic and comparative studies.

This pattern is also seen in Japan, where late‐19th‐ and early‐20th‐century university osteological collections (e.g., the University of Tokyo and Kyoto University) have been used as “modern” reference samples in studies of sex‐ and age‐related, temporal, and regional variation (e.g., Hanihara 1959; Mitsuhashi 1958; Miyamoto 1924a, 1924b, 1924c; Sakaue 2006). However, well‐known secular trends over the last century, such as increases in body size, changes in body proportions, and shifts in health, possibly suggest that these historical collections may not match present‐day populations (Cole 2003; Roche 1979). In the United States, newer donated/documented collections (e.g., the William M. Bass Donated Skeletal Collection; Shirley et al. 2011) make it possible to examine secular change from the 19th to the 21st century, showing clear shifts in cranial size and shape (e.g., Jantz and Meadows Jantz 2000; Kilroy et al. 2020). Jantz and Meadows Jantz (2000) reported that cranial vault dimensions changed noticeably over time, showing increased vault height accompanied by reduced vault breadth and altered vault proportions, whereas facial dimensions shifted modestly. Kilroy et al. (2020) reported that the frequencies of multiple cranial and mandibular nonmetric traits also showed significant temporal change. In Japan, however, a large, widely accessible modern documented skeletal collection has not been available; therefore, late‐19th‐ and early‐20th‐century specimens have continued to serve as the modern reference, and recent secular change in skeletal cranial form has been difficult to evaluate.

Large‐scale somatometric surveys of living Japanese by Kouchi (Kouchi 2000, 2018) have previously shown cohort differences in body size and head dimensions, but measurements on living bodies do not directly capture skeletal cranial shape and cannot replace osteological data. Recently, the growing use of autopsy imaging (Ai) and postmortem computed tomography (PMCT) in Japan allows the creation of a contemporary, well‐documented “virtual skeletal collection.” PMCT provides high‐resolution three‐dimensional (3D) cranial models with associated individual information, making direct, osteology‐based tests of secular change possible.

Accordingly, we apply a 3D geometric morphometric (GM) approach to compare “historical” and “present‐day” Japanese crania, to (1) test for change in cranial shape across the modern era, (2) map the anatomical pattern and direction of that change, and (3) determine whether the pattern and magnitude of sexual dimorphism have changed over the same interval. Based on findings from other populations, we expect an increase in vault height and accompanying shape change over the last century. Clarifying these trends will improve forensic reference standards and help interpret studies that have treated late‐19th‐ and early‐20th‐century series as modern references.

## 2 Materials and Methods

We used computed tomography (CT) scan data from 112 modern Japanese adults, divided into two groups by time period. The historical Japanese group comprised 56 dry crania (34 males and 22 females) from individuals who lived in the late 19th to early 20th century; hereafter, this group is referred to as the “historical” sample. These specimens are housed at the Kyoto University Museum (Kyoto, Japan). The specimens were derived from individuals who died naturally between approximately 1900 and 1920. They were used in anatomical dissection courses at Kyoto University Medical School, prepared primarily as skeletal specimens for research, and subsequently curated as a skeletal collection at the university. For many individuals, the recorded birthplace is Kyoto or one of the nearby prefectures, such as Shiga, Osaka, or Hyogo. Under current institutional guidelines, this historical collection is not considered to raise specific ethical concerns regarding its use for research. Age‐at‐death was available for all specimens except two individuals. In the present dataset, the mean ± standard deviation age‐at‐death was 33.1 ± 12.9 years for males and 37.5 ± 17.9 years for females. The specimens were scanned using a helical‐CT scanner in the Laboratory of Physical Anthropology at Kyoto University for our previous studies (Morita et al. 2014, 2015; Ogihara et al. 2018). The pixel size was 0.468 × 0.468 or 0.5 × 0.5 mm, and the slice thickness was 0.5 mm.

The present‐day Japanese consisted of 56 PMCT scans (29 males and 27 females) of individuals who died between 2022 and 2024; hereafter, this group is referred to as the “present‐day” sample. These data were collected at the Tsukuba Autopsy Center (Tsukuba, Japan). The pixel size was 0.468 × 0.468 mm, and the slice thickness was 1.0 mm. Regarding the use of recent human PMCT data, the experimental protocol was reviewed and approved by the institutional review boards of the Tsukuba Medical Center (no. 2023‐005) and the Office for Life Science Research Ethics and Safety at the University of Tokyo (no. 24‐019). All specimens were free of obvious congenital abnormalities, pathological changes, bone injuries, and surgical scars. Individuals with missing teeth (excluding third molars) due to hypodontia or orthodontic treatment were not included in the present samples. The mean age‐at‐death of the present‐day Japanese dataset was 55.5 ± 19.4 years for males and 55.3 ± 19.6 years for females.

**FIGURE 1:**
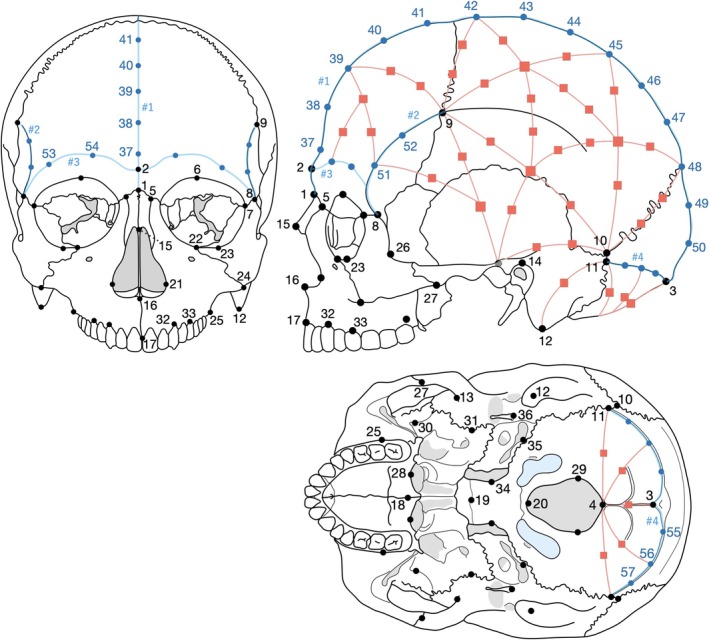
Definitions of all 161 landmarks. Black dots indicate anatomical landmarks (62 points), blue dots indicate equally spaced points along the curves (28 points), and orange squares indicate sliding landmarks (71 points). The orange lines indicate the shortest paths connecting pairs of the anatomical and semilandmarks. Points equally spaced along the calculated shortest paths were defined as the sliding landmarks on the template cranium. Illustrations were modified from Langley et al. (2016).

The CT image data were transferred into medical imaging software (Mimics 26.0; Materialise, Belgium), and 3D surface models of the crania were extracted as triangular mesh models. On the ectocranial surface of each model, we digitized 3D coordinates of 62 anatomical landmarks using reverse engineering software (Geomagic Design X v2024.3; Oqton, Belgium). In addition, a number of points were digitized along four paths (1: midsagittal line, 2: temporal line, 3: supraorbital line, 4: superior nuchal line; Figure 1). These curves were approximated with seventh‐order (Path #1) or fifth‐order (Paths #2–4) Bézier curves, and a total of 28 equally spaced points along the curves were defined as additional landmarks. Points placed along anatomical curves were generated as equidistant points between clearly defined curve endpoints anchored by fixed anatomical landmarks (Morita et al. 2013). Because these curves follow the same anatomical features across specimens, arc‐length parameterization between homologous endpoints provides a consistent correspondence of points along each curve. We therefore used the equidistant curve points as defined, without sliding.

In addition, 71 sliding semi‐landmarks were manually defined on one surface model, selected as the template specimen, to fully represent the cranial shape. This semi‐landmark configuration was mapped to all other crania using a thin‐plate spline function, and the landmarks were subsequently allowed to slide in order to minimize bending energy between each specimen and the template (sliding landmarks) (Gunz et al. 2005). We used sliding semi‐landmarks to represent anatomically corresponding surfaces that lack sufficient discrete anatomical landmarks, thereby establishing geometric correspondence across specimens. These calculations were performed with statistical software (R v4.4; R Foundation for Statistical Computing, Austria) and its packages (Morpho and Rvcg [Schlager 2017], Rsolnp [Ghalanos and Theussl 2015; Ye 1987], and NLopt [Johnson 2008]). In total, each cranium was represented by 161 landmarks (90 nonsliding and 71 sliding) (Figure 1, Table 1).

**TABLE 1:** The descriptions of landmarks.

Point ID	Landmark name	Type	Total points
1	Nasion	m	1
2	Glabella	m	1
3	Inion	m	1
4	Opisthion	m	1
5	Maxillonasofrontale	b	2
6	Most superior point of the part of the supraorbital margin that is lateral to the supraorbital foramen/notch	b	2
7	Frontomalare orbitale	b	2
8	Frontomalare temporale	b	2
9	Stephanion	b	2
10	Asterion	b	2
11	Intersection of the superior nuchal line and the occipitomastoid suture	b	2
12	Mastoidale	b	2
13	Most posterior point of the medial inferior margin of the zygomatic arch	b	2
14	Porion	b	2
15	Rhinion	m	1
16	Akanthion	m	1
17	Prosthion	m	1
18	Alveolon	m	1
19	Sphenobasion	m	1
20	Basion	m	1
21	Alare	b	2
22	Zygoorbitale	b	2
23	Orbitale	b	2
24	Zygomaxillare	b	2
25	Ektomolare	b	2
26	Jugale	b	2
27	Most inferior point of the temporozygomatic suture	b	2
28	Most anterior point of the posteior margin of the palate	b	2
29	Most lateral point of the margin of the foramen magnum	b	2
30	Infratemporale	b	2
31	Stenion	b	2
32	Midpoint of the labial inferior margin of the canine alveolus	b	2
33	Midpoint of the labial inferior margin of the second premolar alveolus	b	2
34	Most medial point of the margin of the foramen lacerum	b	2
35	Most medial point of the posterior margin of the part of the jugular foramen through which the internal jugular vein passes	b	2
36	Most posterior point of the base of the styloid process	b	2
37–50	14 points equally spaced along the midsagittal line (Path #1)	m	14
51–52	2 points equally spaced along the temporal line (Path #2)	b	4
53–54	2 points equally spaced along the supraorbital line (Path #3)	b	4
55–57	3 equally spaced points along the superior nuchal line (Path #4)	b	6
—	Sliding landmarks	—	71
Total			161

Abbreviations: b, bilateral landmark; m, midsagittal landmark.

The coordinates of the 161 landmarks were normalized by centroid size (CS) and registered using generalized Procrustes analysis (GPA) to remove variation due to uniform scaling (isometric size variation), translation, and orientation. Principal component analysis (PCA) was then conducted to obtain the principal components (PCs) of shape variation among the specimens. We analyzed cranial morphological variation using PCA in two ways: (1) a whole‐sample PCA including both historical and present‐day Japanese, to capture the overall trend of cranial shape variation across periods; and (2) separate PCAs for historical or present‐day Japanese, to illustrate differences in the patterns of sexual dimorphism between the two time periods. Cranial shape variations along the PCs were visualized by warping the landmark‐based wireframe from the mean cranial shapes.

We summarized shape variation using PC scores from Procrustes‐aligned coordinates, which remove differences in overall size. Our main question was whether shape differs between time periods (historical vs. present‐day) and between sexes. Therefore, as the primary analysis, we tested the effects of TimePeriod and Sex on the set of PC scores using MANOVA (including the TimePeriod × Sex interaction when relevant). Because CS and age‐at‐death can differ between groups, we treated log_10_ CS and age as secondary variables and checked robustness by repeating the analyses with these covariates included (i.e., MANCOVA). When the multivariate test was significant, we ran follow‐up tests for each PC to identify which axes contributed to the overall effect, applying Bonferroni correction across PCs.

As a sensitivity analysis, we repeated the multivariate tests while varying the number of retained PCs to confirm that the statistical conclusions and the main qualitative patterns relevant to our interpretations were consistent across the choice of the PCs. These calculations were performed in R using the Morpho, Rvcg, and geomorph packages (Adams et al. 2024; Baken et al. 2021).

Measurement error was assessed by repeated digitization of a subset of specimens. Specifically, four specimens (one from each group) were digitized twice by the same observer (SU). The mean digitizing error was 1.25 ± 1.02 mm (mean ± SD). Across all landmarks, mean intergroup displacement was 3.81 ± 1.32 mm (secular change), and 3.56 ± 0.97 mm (sexual dimorphism). Among the top 10% of most displaced landmarks, mean differences reached 6.31 ± 0.71 mm (max: 8.12 mm) and 5.37 ± 0.42 mm (max: 6.15 mm), respectively. Thus, the digitizing error (1.25 mm) is considerably smaller than the observed biological differences. This repeatability assessment was based on a limited subset of specimens; however, the estimated digitizing error was small relative to the interindividual variation captured by the leading PCs, and we therefore consider it unlikely to affect the main conclusions.

To quantify how well sex can be predicted from shape variation, we performed a linear discriminant analysis (LDA; equivalent to DFA under equal covariance assumptions) using the first eight PC scores (PC1–PC8) from the whole‐sample PCA as predictors. Classification performance was evaluated by leave‐one‐out cross‐validation, where the discriminant function was trained on *n* − 1 individuals (*n* denotes the number of specimens) and tested on the held‐out individual, repeated for all individuals. We report overall accuracy, balanced accuracy, and the confusion matrix. These analyses were conducted using the MASS package (Venables and Ripley 2002) in R.

In this study, we analyzed the skull as a single structure because our primary goal was to characterize global patterns of cranial shape variation and integration across the entire skull. Although the skull can be subdivided into facial, neurocranial, and basicranial regions, our primary aim was to characterize global cranial shape variation across the entire skull. Region‐specific analyses can be informative and may reduce dimensionality, but they would shift the focus from whole‐skull patterns and introduce additional analytical choices and multiple comparisons. We therefore used a whole‐skull landmark configuration for the main GM analyses and interpreted our results as overall cranial shape patterns rather than region‐specific modular responses.

In GM, it is common that the number of Procrustes shape variables exceeds the number of specimens. In such high‐dimensional settings, multivariate methods that require inversion of the variance–covariance matrix of Procrustes coordinates (e.g., CVA) are unreliable or impossible to compute, because a stable matrix inverse requires more cases than variables, a condition that is often not met in modern morphometrics (Mitteroecker and Gunz 2009). By contrast, PCA of Procrustes coordinates is mathematically well defined and provides an exploratory low‐dimensional summary of shape variation. Because the sample covariance matrix is estimated from *n* specimens, it can have at most *n* − 1 independent dimensions; accordingly, only the first *n* − 1 PCs can have nonzero eigenvalues. We therefore focused our interpretation on the leading PCs and treated higher‐order PCs cautiously, as they can be more sensitive to sampling noise when sample sizes are limited (Cardini and Elton 2007; Cardini et al. 2015).

In addition to these statistical considerations, methodological issues related to landmark placement and visualization also warrant attention. It has been noted that equidistant placement of curve points can introduce unwanted variance as an artifact if the underlying curve is poorly defined, for example when it crosses sharp corners or other characteristic features (Gunz et al. 2005). To minimize this risk, we defined curves to follow anatomically meaningful trajectories and avoided passing sharp corners or other distinctive features. More generally, semilandmarks are not discrete anatomical loci; although they provide a practical representation of curves and surfaces, their correspondence is established geometrically and does not, by itself, guarantee biological homology. In addition, Procrustes superimposition can sometimes make the movement of individual points look larger or more widespread in visualizations (the “Pinocchio effect”; Klingenberg 2021). For these reasons, we focus on clear, large‐scale regional patterns and avoid over‐interpreting small, localized differences that may depend on semilandmark placement or density (Klingenberg 2021).

## 3 Results

Age‐at‐death differed significantly between the two time periods, with the present‐day sample being older on average by approximately 20 years (*p* < 0.001). In addition, CS was significantly larger in males than in females in both time periods (*p* < 0.001 in both cases), and CS was significantly larger in the present‐day sample than in the historical sample within each sex (*p* < 0.001 for both comparisons). These results indicate that both age and overall size may confound group comparisons based on PC scores; therefore, PC score differences should be analyzed while statistically controlling for age‐at‐death and CS.

Shape variation was analyzed using PCA of the Procrustes‐aligned coordinates. For statistical analyses, we retained PC1–PC8, based on the scree plot and the criterion that each retained PC explained at least 3% of the total variance (Figure S1, Table S1). Therefore, when the multivariate test was significant, follow‐up tests on individual PCs were evaluated using an adjusted significance threshold of *α* = 0.05/8.

Using this PC score set (PC1–PC8), we first evaluated whether age‐at‐death and CS contributed to shape variation using a MANCOVA on PC scores (PC1–PC8), with TimePeriod, Sex, age‐at‐death, and log_10_ CS as predictors. In this model, age‐at‐death was not significant (*p* = 0.49, n.s.). Therefore, age was not treated as a key explanatory variable in the analyses below (Table S2). In contrast, CS differed systematically between groups. log_10_ CS was significantly different between time periods (*p* < 0.001) and between sexes (*p* < 0.001), with no evidence of a TimePeriod × Sex interaction (*p* = 0.16, n.s.) (Table S3). This indicates that size variation is associated with TimePeriod and Sex.

**FIGURE 2:**
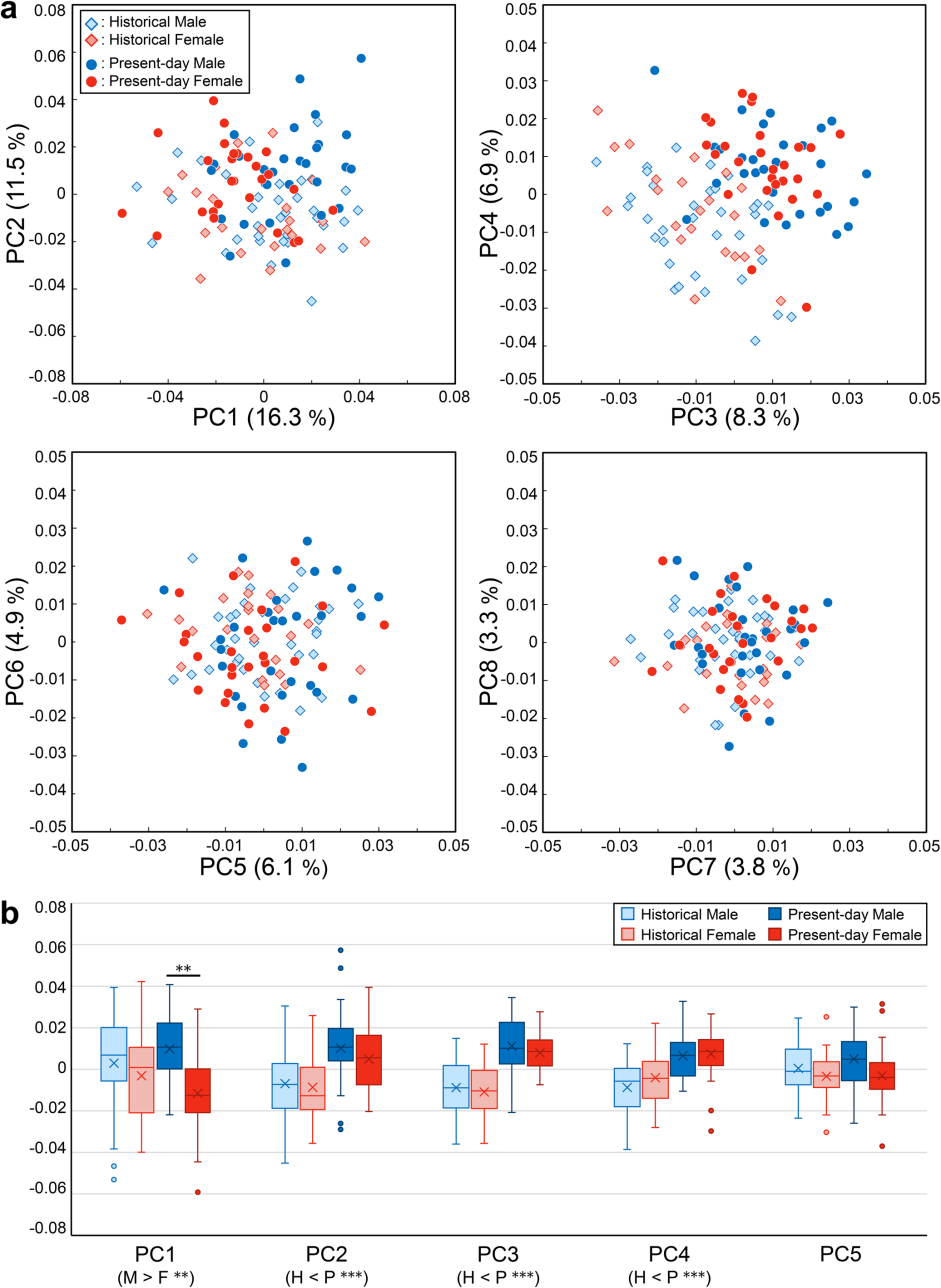
Results of the whole‐sample principal component analysis (PCA). (a) Scatter plots of the scores for the first eight principal components (PCs). Diamonds: historical; circles: present‐day; blue: male; red: female. (b) Box‐and‐whisker plots of the scores for the first five PCs. The horizontal line within each box indicates the median, and the cross indicates the mean. Boxes extend from the 25th to 75th percentiles, with whiskers spanning the minimum and maximum values, excluding outliers (dots: values > 1.5 interquartile ranges from the box). Light blue: historical male; light red: historical female; dark blue: present‐day male; dark red: present‐day female. *p < 0.05/8, **p < 0.01/8, ***p < 0.001/8. M: male (historical male + present‐day male), F: female, H: historical (historical male + historical female), P: present‐day.

The results of the whole‐sample PCA are presented in Figure 2a as scatter plots of PC1 versus PC2, PC3 versus PC4, PC5 versus PC6, and PC7 versus PC8. The first eight PCs accounted for 61.1% of the total variance (PC1: 16.3%, PC2: 11.5%, PC3: 8.3%, PC4: 6.9%, PC5: 6.1%, PC6: 4.9%, PC7: 3.8%, PC8: 3.3%). All remaining components explained less than 3.0% each of the total variation (Table S1). MANOVA using the first eight PCs detected statistically significant overall differences both between the two periods (Pillai's trace = 0.88, *F* = 93.18, *p* < 0.001) and between the sexes (Pillai's trace = 0.19, *F* = 2.88, *p* = 0.0063), with no interaction observed between sex and period (Pillai's trace = 0.11, *F* = 1.51, *p* = 0.16) (Table S4). According to the *R* ^2^ value for the correlation between CS and PC scores, CS accounts for 4.2%, 1.4%, 20.4%, 2.1%, 4.7%, 0.2%, 6.2%, and 1.0% of the shape variance expressed by PC1–PC8, respectively. These results indicate that, overall, shape differs between time periods and also differs between sexes.

ANOVA on the individual PC scores revealed that values in the present‐day Japanese were significantly larger than those in the historical Japanese for PC2, PC3, and PC4 (each *p* < 0.001). In contrast, male values were significantly larger than female values for PC1 (*p* < 0.001) (Figure 2b, Table S5). Therefore, PC1 primarily reflects sexual dimorphism, whereas PC2, PC3, and PC4 correspond to temporal differences between the two periods. In addition, present‐day male PC1 scores were significantly larger than present‐day female scores (*p* < 0.001), while no significant difference was observed between historical male values and historical female values. This indicates that the sexual dimorphism of modern Japanese crania has increased over time (Figure 2b).

**FIGURE 3:**
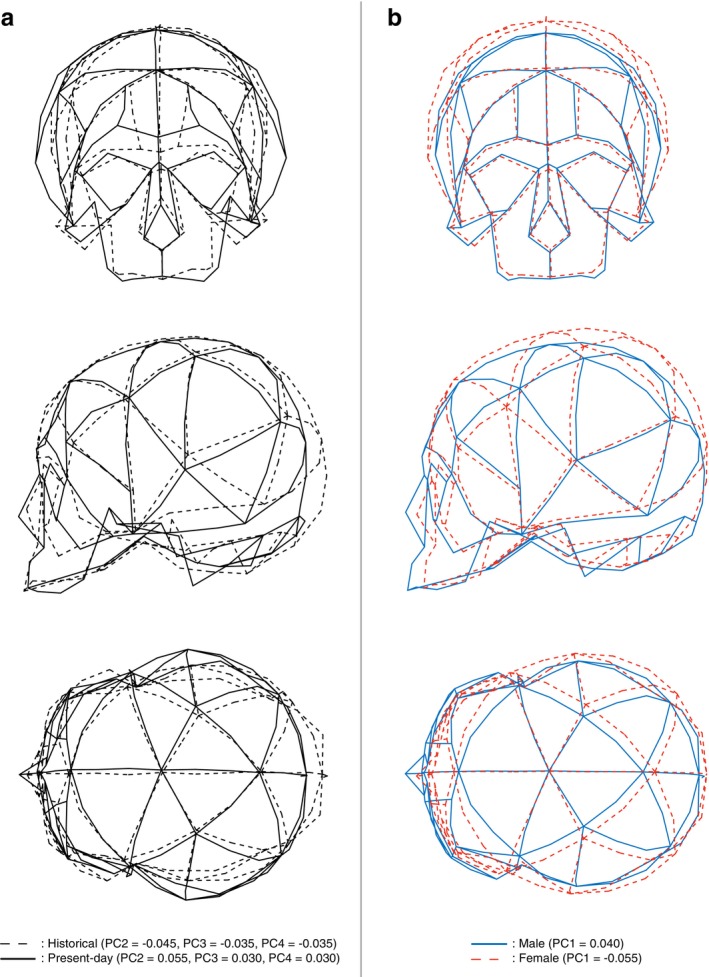
Shape variation along the PCs in the whole‐sample PCA. (a) Cranial shape variation between historical and present‐day populations. PC scores that differed significantly between the two periods were modified from the mean shape to generate more typical historical (dashed lines; PC2 = −0.045, PC3 = −0.035, PC4 = −0.035) and present‐day (solid lines; PC2 = 0.055, PC3 = 0.030, PC4 = 0.030) shapes. Top: coronal view; middle: sagittal view; bottom: axial (superior) view. (b) Cranial shape variation between sexes. PC scores that differed significantly between males and females were modified to generate more masculine (solid blue lines; PC1 = 0.040) and more feminine (dashed red lines; PC1 = −0.055) shapes.

Figure 3a illustrates morphological changes between the two periods by reconstructing shapes from the mean shape model while varying only the scores along PCs that differed significantly between groups (PC2–PC4), with all other PC scores held at zero (i.e., at the overall mean shape). The cranial shape typical of present‐day Japanese (solid black line) tended to be wider in the transverse dimension and shorter anteroposteriorly than the shape typical of historical Japanese (dashed black line), indicating brachycephalization in the modern population. In particular, the neurocranium is wider due to a relative elongation of cranial breadth, shorter owing to a relative anteroposterior contraction of the parietal bone, and lower as a result of a relative decrease in cranial height, with the cranial base shifted superiorly. The mastoid process is larger and more projecting. The nasal bone shows a tendency to protrude more anteriorly while becoming narrower in width. The supraorbital line is located more superiorly, and the forehead appears more concave. In addition, the bizygomatic width is reduced, whereas the maxilla is relatively wider.

Figure 3b illustrates wireframes representing male (solid blue line) and female (dashed red line) cranial shapes generated by modifying PC scores that showed significant sex differences (PC1). Male crania, particularly in the modern era, exhibit a number of distinctive features. The supraorbital ridge is more prominent, and the forehead slopes backward in a more horizontal fashion. The parieto‐occipital region is positioned more anteroinferiorly, giving the cranial vault a slightly different profile. The face projects more anteriorly, with both the glabellar and midfacial regions becoming more protruding. The zygomatic bones are more strongly developed. The maxilla is larger and projects more prominently, while the nasal bone is both larger and more protruding. The mastoid process is enlarged and more prominent compared to females.

**FIGURE 4:**
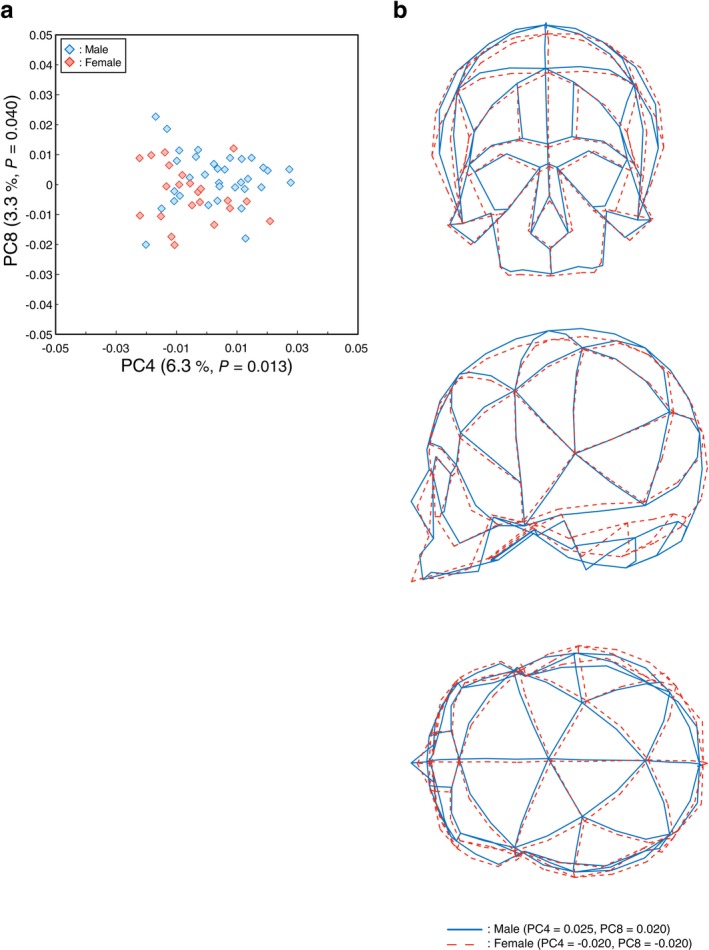
Sexual dimorphism in the historical population. (a) Scatter plot of the PCA results for historical samples only (blue: male; red: female). *P* values in parentheses indicate ANOVA results. (b) Cranial shape variation between sexes in the historical population. PC scores that tended to differ between males and females were modified from the mean shape to generate typical male (solid blue lines; PC4 = 0.025, PC8 = 0.020) and female (dashed red lines; PC4 = −0.020, PC8 = −0.020) shapes. Top: coronal view; middle: sagittal view; bottom: axial (superior) view.

**FIGURE 5:**
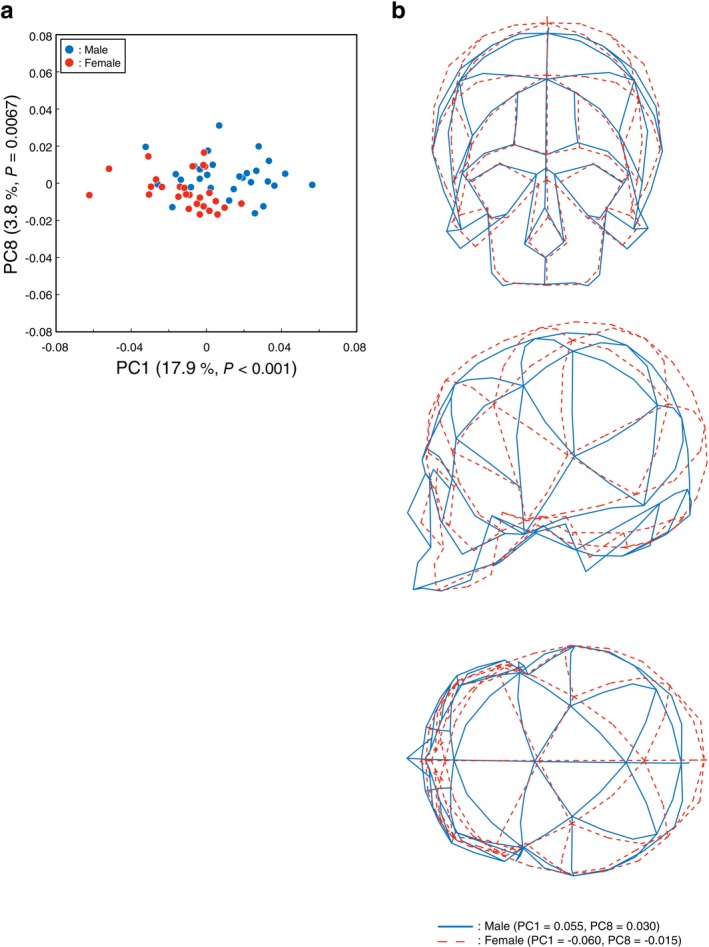
Sexual dimorphism in the present‐day population. (a) Scatter plot of the PCA results for present‐day samples only (blue: male; red: female). *P* values in parentheses indicate ANOVA results. (b) Cranial shape variation between sexes in the present‐day population. PC scores that tended to differ between males and females were modified to generate typical male (solid blue lines; PC1 = 0.055, PC8 = 0.030) and female (dashed red lines; PC1 = −0.060, PC8 = −0.015) shapes. Top: coronal view; middle: sagittal view; bottom: axial (superior) view.

The separate PCAs for historical or present‐day Japanese were then conducted to explore possible secular trends in sexual dimorphism. In PCA of historical specimens (Figure 4a), the first nine PCs accounted for 69.6% of the total variance (PC1: 21.2%, PC2: 12.4%, PC3: 10.6%, PC4: 6.3%, PC5: 4.8%, PC6: 4.5%, PC7: 3.7%, PC8: 3.3%, PC9: 3.0%). MANOVA using the first nine PC scores indicated marginally significant sex differences were detected in the historical group (Pillai's trace = 0.29, *F* = 2.04, *p* = 0.055). ANOVA revealed that none of the PCs showed a statistically significant sex effect after Bonferroni correction (*α* = 0.05/9). However, PC4 and PC8 tended to be larger in males than in females, and these two PCs showed the smallest *p*‐values among the tested PCs (PC4: *p* = 0.013; PC8: *p* = 0.040) (Table S6). We therefore treat these results as suggestive (trend‐level) evidence of sexual dimorphism and explore them further in the subsequent analyses, while interpreting them cautiously given the multiple‐testing correction. According to the *R* ^2^ values, CS accounts for 6.1%, 2.4%, 2.9%, 9.9%, 1.7%, 0.4%, 0.7%, 9.0%, and 2.9% of the shape variance expressed by PC1–PC9, respectively. In PCA of present‐day specimens (Figure 5a), the first nine PCs accounted for 67.7% of the total variance (PC1: 17.9%, PC2: 11.7%, PC3: 8.6%, PC4: 6.8%, PC5: 6.5%, PC6: 4.6%, PC7: 4.4%, PC8: 3.8%, PC9: 3.3%). MANOVA indicated there exists a significant sex difference (Pillai's trace = 0.58, *F* = 7.18, *p* < 0.001), and ANOVA revealed that PC1 (*p* < 0.001) was significantly larger in males than in females, and PC8 was also larger in males, showing a marginal (trend‐level) effect (*p* = 0.0067) (Table S7). We therefore focused on PC1 and PC8 in the subsequent analyses. CS accounts for 5.6%, 2.1%, 5.5%, 6.4%, 3.9%, 0.8%, 0.2%, 9.8%, and 1.4% of the shape variance expressed by PC1–PC9, respectively.

Figures 4b and 5b illustrate wireframes representing sexual differences between male (solid blue line) and female (dashed red line) cranial shapes in the historical and present‐day Japanese groups, respectively. In the historical group, the degree of sexual difference was relatively low, but in males, it was observed that the superior surface of the frontal bone was positioned more superiorly, the mastoid process projected more inferiorly, and the portion of the occipital squama above and below the superior nuchal lines projected more inferoposteriorly (Figure 4b). In the present‐day group, the pattern of sexual difference was generally similar to that shown in Figure 3b (Figure 5b).

We assessed how well sex could be discriminated from the first eight PC scores using a LDA with leave‐one‐out cross‐validation. Classification accuracy was 67.0% for the whole‐sample PCA. Balanced accuracy was 66.3%, and the confusion matrix is shown in Table S8. This performance exceeded the majority‐class baseline (56.3%), indicating above‐chance discrimination.

We performed sensitivity analyses to check that our results were not driven by analytic choices. Changing the number of retained PCs (PC1–PC5, PC1–PC8, PC1–PC10, and PC1–PC12) did not affect the main conclusions: the effects of TimePeriod and Sex remained consistent across specifications. In contrast, the TimePeriod × Sex interaction was not consistent: it was not significant for PC1–PC5 or PC1–PC8 but became only nominally significant when additional low‐variance PCs were included (PC1–PC10 and PC1–PC12; *p* = 0.036 and 0.044) (Table S9). Given the small margin and the fact that multiple PC‐retention choices were tested, we do not consider this interaction statistically significant and base our main conclusions on the pre‐specified PC set (PC1–PC8).

## 4 Discussion

The present study of morphological analysis of Japanese crania using GM clearly demonstrated that there are clear secular changes in cranial shape over the past century. We found that present‐day Japanese tend to exhibit more brachycephalic features, with a higher cranial base, a larger mastoid process, a more protruding but narrower nasal bone, a superiorly located supraorbital line, a concave forehead, narrower bizygomatic width, and a wider maxilla, relative to the historical Japanese group (Figure 3a). The recent trend of brachycephalization has been documented previously in Japan (Kouchi 2000, 2018), and analyses of nonmetric traits have suggested that secular changes occur primarily in the nasal and zygomatic regions (Kilroy et al. 2020). Our results are generally consistent with those reports. It should be noted, however, that a recent trend of de‐brachycephalization has been documented in the United States (Jantz and Meadows Jantz 2000), which possibly in part reflect the heterogeneous genetic backgrounds resulting from large‐scale immigration in the United States. However, these previous studies were based mainly on a limited number of linear measurements such as cranial length. The present study is the first to demonstrate detailed 3D secular changes of the cranium over the past century in modern Japanese.

Because age‐at‐death and overall size are known to influence cranial morphology, we explicitly evaluated their potential confounding effects. In our sample, age‐at‐death differed between the two time periods. This difference is expected given secular changes in longevity and demographic structure over the past century. However, age‐at‐death was not a significant predictor of the retained PC scores (PC1–PC8) in the MANCOVA, suggesting that the main secular patterns described here are not readily explained by age alone in the analyzed PC space. Nevertheless, age and time period are partially confounded in historical versus present‐day collections, and residual confounding cannot be completely excluded; future work using age‐matched subsets or independent collections will further strengthen causal interpretation. In contrast, CS differed systematically by sex and time period. This indicates that secular and sex‐related differences in cranial size coexist with shape differences; accordingly, we interpret the observed shape patterns while recognizing that allometry may contribute to, but does not necessarily fully account for, the reported secular trends.

In addition, the present study revealed that sexual dimorphism in Japanese cranial morphology was relatively small about 100 years ago but has become more pronounced over the past century. This suggests that sex determination is more feasible in present‐day populations and also indicates the need to update discriminant functions for sex estimation. However, this does not suggest the emergence of any new sexually dimorphic traits. The present study rather confirms the presence of features that have already been described in earlier studies. Male crania, particularly in the modern era, tended to exhibit a more prominent supraorbital ridge, a forehead sloping backward more horizontally, a face projecting more anteriorly with a more protruding glabellar and midfacial region, more pronounced zygomatic bones, a larger and more projecting maxilla, a larger and more protruding nasal bone, and a more prominent mastoid process (Figures 3, 4, 5), all of which have been documented previously (Abushehab et al. 2024; Acsadi and Nemeskeri 1970; Ferembach et al. 1980; Ingerslev and Solow 1975; Krogman and İşcan 1986; Rogers 1991, 2005; White et al. 2011).

Our LDA with leave‐one‐out cross‐validation achieved only moderate discrimination (balanced accuracy = 66%), indicating substantial overlap between sexes and limiting its use as a definitive tool for individual‐level sex estimation. Nonetheless, performance above the majority‐class baseline supports that sex‐related shape differences are detectable at the group level, consistent with the multivariate tests and the wireframe visualizations.

Although the exact causes of these changes, both the temporal differences and the increase in sexual dimorphism, are obscure, they may be related to well‐known secular trends over the last century, such as increases in body size, changes in body proportions, and improvements in health due to better nutrition (Cole 2003; Roche 1979). It is also possible that dietary changes, such as greater consumption of soft foods and the resulting reduction in chewing load, have contributed to modifications of the facial skeleton and mandible. Future research will be necessary to investigate the underlying causes of these changes in detail.

The present study was designed to document secular and sex‐related cranial shape changes specifically in Japanese individuals. Accordingly, our inferences are limited to this population, and whether the observed patterns represent broader, global tendencies in recent *Homo sapiens* or are region‐specific remains to be evaluated through cross‐population comparisons. In addition, although GM allowed us to capture detailed cranial morphology, the available skeletal collections may not fully reflect the entire variation of the population. Examining these detailed patterns of change is important for understanding the relationship between biology and environment, the influence of nutrition, health, and lifestyle on cranial morphology, and the ways in which human skeletal form continues to change under modern living conditions.

## Author Contributions

**Shiori Usui:** conceptualization, methodology, investigation, writing – original draft, writing – review and editing, data curation, formal analysis, visualization, software. **Hideki Amano:** methodology, investigation, writing – review and editing, software. **Hideyuki Hayakawa:** data curation, writing – review and editing, investigation. **Seiji Shiotani:** data curation, writing – review and editing, investigation. **Naomichi Ogihara:** writing – original draft, writing – review and editing, conceptualization, methodology, supervision, project administration, investigation.

## Ethics Statement

Historical Japanese specimens housed at the Kyoto University Museum derive from individuals who died naturally between approximately 1900 and 1920. Under current institutional guidelines, this historical collection is not considered to raise specific ethical concerns regarding its use for research. Regarding the use of recent human postmortem computed tomography (PMCT) data, the experimental protocol was reviewed and approved by the institutional review boards of the Tsukuba Medical Center and the Office for Life Science Research Ethics and Safety at the University of Tokyo. We only used PMCT data for which consent to use for research purposes had been obtained from bereaved families.

## Conflicts of Interest

The authors declare no conflicts of interest.

## Supporting information


**Figure S1:** Scree plot of the percentage of variance explained in the whole‐sample PCA.
**Table S1:** Eigenvalue, variance explained, and cumulative variance explained for each PC in the whole‐sample PCA (*N* = 112).
**Table S2:** Results of the MANCOVA on PC1–PC8 scores from the whole‐sample PCA including TimePeriod, Sex, age‐at‐death, and log_10_ CS (*N* = 110).
**Table S3:** Results of the ANOVA on log_10_ CS including TimePeriod and Sex (*N* = 110).
**Table S4:** Results of the MANOVA on PC1–PC8 scores from the whole‐sample PCA including TimePeriod and Sex (*N* = 112).
**Table S5:** Results of the ANOVA on PC1–PC8 scores from the whole‐sample PCA including TimePeriod and Sex (*N* = 112).
**Table S6:** Results of the ANOVA on PC1–PC9 scores from the separate PCA for the historical samples including Sex (*N* = 56).
**Table S7:** Results of the ANOVA on PC1–PC9 scores from the separate PCA for the present‐day samples including Sex (*N* = 56).
**Table S8:** Confusion matrix for the LDA with leave‐one‐out cross‐validation.
**Table S9:** Results of the sensitivity analyses.

## Data Availability

The data that support the findings of this study, except for the CT scan images, are available from the corresponding author upon reasonable request. The CT scan data used in this study are subject to restrictions due to participant privacy concerns and ethical restrictions.
