# Supplementary material for: Secular Changes in Cranial Morphology and Pattern of Sexual Dimorphism in Modern Japanese: A Geometric Morphometric Analysis Using Post‐Mortem Computed Tomography Data
Source: Am J Biol Anthropol. 2026 Apr 3;189(4):e70235. doi: 10.1002/ajpa.70235 (PMC13047470; doi:10.1002/ajpa.70235)
Supplement: Supplementary file 1 — Figure S1: Scree plot of the percentage of variance explained in the whole‐sample PCA. Table S1: Eigenvalue, variance explained, and cumulative variance explained for each PC in the whole‐sample PCA (N = 112). Table S2: Results of the MANCOVA on PC1–PC8 scores from the whole‐sample PCA including TimePeriod, Sex, age‐at‐death, and log10 CS (N = 110). Table S3: Results of the ANOVA on log10 CS including TimePeriod and Sex (N = 110). Table S4: Results of the MANOVA on PC1–PC8 scores from the whole‐sample PCA including TimePeriod and Sex (N = 112). Table S5: Results of the ANOVA on PC1–PC8 scores from the whole‐sample PCA including TimePeriod and Sex (N = 112). Table S6: Results of the ANOVA on PC1–PC9 scores from the separate PCA for the historical samples including Sex (N = 56). Table S7: Results of the ANOVA on PC1–PC9 scores from the separate PCA for the present‐day samples including Sex (N = 56). Table S8: Confusion matrix for the LDA with leave‐one‐out cross‐validation. Table S9: Results of the sensitivity analyses. [file AJPA-189-e70235-s001.docx]

**Secular changes in cranial morphology and pattern of sexual dimorphism in modern Japanese: A geometric morphometric analysis using post-mortem computed tomography data**

Shiori Usui^1,2†^, Hideki Amano^1^, Hideyuki Hayakawa^3^, Seiji Shiotani^4^, and Naomichi Ogihara^1†^

1) Laboratory of Human Evolutionary Biomechanics, Department of Biological Sciences, Graduate School of Science, The University of Tokyo, 7-3-1, Hongo, Bunkyo-ku, Tokyo 113-0033, Japan.
2) Second Biology Section, First Department of Forensic Science, National Research Institute of Police Science, 6-3-1, Kashiwanoha, Kashiwa-shi, Chiba 277-0882, Japan.
3) Department of Forensic Medicine, Tsukuba Medical Examiner’s Office, 1-3-1, Amakubo, Tsukuba-shi, Ibaraki 305-8558, Japan.
4) Department of Radiology, Seirei Fuji Hospital, 3-1, Minami-cho, Fuji-shi, Shizuoka 417-0026, Japan.

Corresponding Authors^†^

Shiori Usui [usui-shiori@g.ecc.u-tokyo.ac.jp](mailto:usui-shiori@g.ecc.u-tokyo.ac.jp)

Naomichi Ogihara [ogihara@bs.s.u-tokyo.ac.jp](mailto:ogihara@bs.s.u-tokyo.ac.jp)


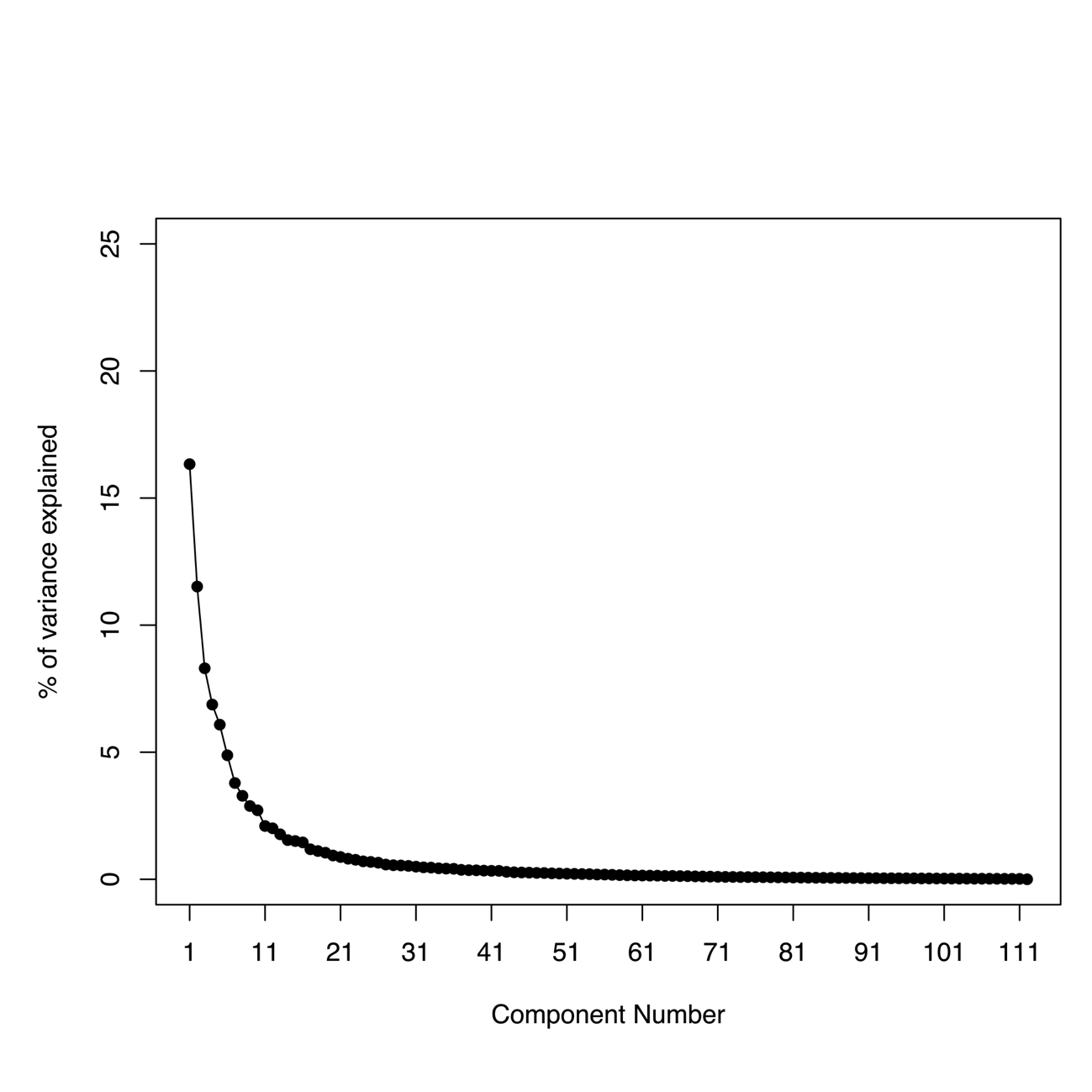
 Supplementary Figure S1. Scree plot of the percentage of variance explained in the whole-sample PCA.

Supplementary Table S1. Eigenvalue, variance explained, and cumulative variance explained for each PC in the whole-sample PCA (*N* = 112).

|  | PC1 | PC2 | PC3 | PC4 | PC5 | PC6 | PC7 | PC8 | PC9 | PC10 |
| --- | --- | --- | --- | --- | --- | --- | --- | --- | --- | --- |
| Eigen value | 4.78×10^-4^ | 3.37×10^-4^ | 2.43×10^-4^ | 2.01×10^-4^ | 1.78×10^-4^ | 1.43×10^-4^ | 1.11×10^-4^ | 9.61×10^-5^ | 8.44×10^-5^ | 7.94×10^-5^ |
| Variance explained [%] | 16.33 | 11.52 | 8.30 | 6.88 | 6.08 | 4.88 | 3.79 | 3.28 | 2.88 | 2.71 |
| Cumulative variance explained [%] | 16.33 | 27.85 | 36.15 | 43.03 | 49.11 | 53.99 | 57.78 | 61.07 | 63.95 | 66.66 |

|  | PC11 | PC12 | PC13 | PC14 | PC15 | PC16 | PC17 | PC18 | PC19 | PC20 |
| --- | --- | --- | --- | --- | --- | --- | --- | --- | --- | --- |
| Eigen value | 6.14×10^-5^ | 5.87×10^-5^ | 5.18×10^-5^ | 4.51×10^-5^ | 4.40×10^-5^ | 4.25×10^-5^ | 3.45×10^-5^ | 3.25×10^-5^ | 3.06×10^-5^ | 2.74×10^-5^ |
| Variance explained [%] | 2.10 | 2.01 | 1.77 | 1.54 | 1.50 | 1.45 | 1.18 | 1.11 | 1.05 | 0.94 |
| Cumulative variance explained [%] | 68.76 | 70.76 | 72.53 | 74.08 | 75.58 | 77.03 | 78.21 | 79.32 | 80.37 | 81.31 |

|  | PC21 | PC22 | PC23 | PC24 | PC25 | PC26 | PC27 | PC28 | PC29 | PC30 |
| --- | --- | --- | --- | --- | --- | --- | --- | --- | --- | --- |
| Eigen value | 2.56×10^-5^ | 2.37×10^-5^ | 2.24×10^-5^ | 2.06×10^-5^ | 2.00×10^-5^ | 1.91×10^-5^ | 1.69×10^-5^ | 1.62×10^-5^ | 1.59×10^-5^ | 1.54×10^-5^ |
| Variance explained [%] | 0.88 | 0.81 | 0.77 | 0.71 | 0.68 | 0.65 | 0.58 | 0.55 | 0.54 | 0.53 |
| Cumulative variance explained [%] | 82.18 | 82.99 | 83.76 | 84.46 | 85.15 | 85.80 | 86.38 | 86.93 | 87.47 | 88.00 |

|  | PC31 | PC32 | PC33 | PC34 | PC35 | PC36 | PC37 | PC38 | PC39 | PC40 |
| --- | --- | --- | --- | --- | --- | --- | --- | --- | --- | --- |
| Eigen value | 1.45×10^-5^ | 1.37×10^-5^ | 1.34×10^-5^ | 1.26×10^-5^ | 1.23×10^-5^ | 1.21×10^-5^ | 1.09×10^-5^ | 1.05×10^-5^ | 1.04×10^-5^ | 9.97×10^-6^ |
| Variance explained [%] | 0.50 | 0.47 | 0.46 | 0.43 | 0.42 | 0.41 | 0.37 | 0.36 | 0.35 | 0.34 |
| Cumulative variance explained [%] | 88.49 | 88.96 | 89.42 | 89.85 | 90.27 | 90.69 | 91.06 | 91.42 | 91.77 | 92.11 |

|  | PC41 | PC42 | PC43 | PC44 | PC45 | PC46 | PC47 | PC48 | PC49 | PC50 |
| --- | --- | --- | --- | --- | --- | --- | --- | --- | --- | --- |
| Eigen value | 9.73×10^-6^ | 9.69×10^-6^ | 8.54×10^-6^ | 8.06×10^-6^ | 7.83×10^-6^ | 7.64×10^-6^ | 7.33×10^-6^ | 7.21×10^-6^ | 6.83×10^-6^ | 6.63×10^-^6 |
| Variance explained [%] | 0.33 | 0.33 | 0.29 | 0.28 | 0.27 | 0.26 | 0.25 | 0.25 | 0.23 | 0.23 |
| Cumulative variance explained [%] | 92.44 | 92.77 | 93.07 | 93.34 | 93.61 | 93.87 | 94.12 | 94.37 | 94.60 | 94.83 |

|  | PC51 | PC52 | PC53 | PC54 | PC55 | PC56 | PC57 | PC58 | PC59 | PC60 |
| --- | --- | --- | --- | --- | --- | --- | --- | --- | --- | --- |
| Eigen value | 6.41×10^-6^ | 6.25×10^-6^ | 6.02×10^-6^ | 5.90×10^-6^ | 5.49×10^-6^ | 5.44×10^-6^ | 5.13×10^-6^ | 4.77×10^-6^ | 4.58×10^-6^ | 4.39×10^-6^ |
| Variance explained [%] | 0.22 | 0.21 | 0.21 | 0.20 | 0.19 | 0.19 | 0.18 | 0.16 | 0.16 | 0.15 |
| Cumulative variance explained [%] | 95.05 | 95.26 | 95.47 | 95.67 | 95.85 | 96.04 | 96.22 | 96.38 | 96.54 | 96.69 |

|  | PC61 | PC62 | PC63 | PC64 | PC65 | PC66 | PC67 | PC68 | PC69 | PC70 |
| --- | --- | --- | --- | --- | --- | --- | --- | --- | --- | --- |
| Eigen value | 4.30×10^-6^ | 4.19×10^-6^ | 4.16×10^-6^ | 3.88×10^-6^ | 3.85×10^-6^ | 3.68×10^-6^ | 3.60×10^-6^ | 3.35×10^-6^ | 3.19×10^-6^ | 3.08×10^-6^ |
| Variance explained [%] | 0.15 | 0.14 | 0.14 | 0.13 | 0.13 | 0.13 | 0.12 | 0.11 | 0.11 | 0.11 |
| Cumulative variance explained [%] | 96.83 | 96.98 | 97.12 | 97.25 | 97.38 | 97.51 | 97.63 | 97.75 | 97.85 | 97.96 |

|  | PC71 | PC72 | PC73 | PC74 | PC75 | PC76 | PC77 | PC78 | PC79 | PC80 |
| --- | --- | --- | --- | --- | --- | --- | --- | --- | --- | --- |
| Eigen value | 2.86×10^-6^ | 2.77×10^-6^ | 2.72×10^-6^ | 2.60×10^-6^ | 2.54×10^-6^ | 2.48×10^-6^ | 2.41×10^-6^ | 2.32×10^-6^ | 2.20×10^-6^ | 2.11×10^-6^ |
| Variance explained [%] | 0.10 | 0.09 | 0.09 | 0.09 | 0.09 | 0.08 | 0.08 | 0.08 | 0.08 | 0.07 |
| Cumulative variance explained [%] | 98.06 | 98.15 | 98.25 | 98.33 | 98.42 | 98.51 | 98.59 | 98.67 | 98.74 | 98.81 |

|  | PC81 | PC82 | PC83 | PC84 | PC85 | PC86 | PC87 | PC88 | PC89 | PC90 |
| --- | --- | --- | --- | --- | --- | --- | --- | --- | --- | --- |
| Eigen value | 2.04×10^-6^ | 1.93×10^-6^ | 1.83×10^-6^ | 1.74×10^-6^ | 1.68×10^-6^ | 1.65×10^-6^ | 1.56×10^-6^ | 1.50×10^-6^ | 1.43×10^-6^ | 1.38×10^-6^ |
| Variance explained [%] | 0.07 | 0.07 | 0.06 | 0.06 | 0.06 | 0.06 | 0.05 | 0.05 | 0.05 | 0.05 |
| Cumulative variance explained [%] | 98.88 | 98.95 | 99.01 | 99.07 | 99.13 | 99.19 | 99.24 | 99.29 | 99.34 | 99.39 |

|  | PC91 | PC92 | PC93 | PC94 | PC95 | PC96 | PC97 | PC98 | PC99 | PC100 |
| --- | --- | --- | --- | --- | --- | --- | --- | --- | --- | --- |
| Eigen value | 1.29×10^-6^ | 1.27×10^-6^ | 1.18×10^-6^ | 1.16×10^-6^ | 1.11×10^-6^ | 1.09×10^-6^ | 1.06×10^-6^ | 9.69×10^-7^ | 9.31×10^-7^ | 8.94×10^-7^ |
| Variance explained [%] | 0.04 | 0.04 | 0.04 | 0.04 | 0.04 | 0.04 | 0.04 | 0.03 | 0.03 | 0.03 |
| Cumulative variance explained [%] | 99.43 | 99.47 | 99.51 | 99.55 | 99.59 | 99.63 | 99.66 | 99.70 | 99.73 | 99.76 |

|  | PC101 | PC102 | PC103 | PC104 | PC105 | PC106 | PC107 | PC108 | PC109 | PC110 | PC111 |
| --- | --- | --- | --- | --- | --- | --- | --- | --- | --- | --- | --- |
| Eigen value | 8.19×10^-7^ | 8.00×10^-7^ | 7.36×10^-7^ | 7.14×10^-7^ | 6.59×10^-7^ | 6.52×10^-7^ | 6.14×10^-7^ | 5.83×10^-7^ | 5.10×10^-7^ | 4.98×10^-7^ | 4.28×10^-7^ |
| Variance explained [%] | 0.03 | 0.03 | 0.03 | 0.02 | 0.02 | 0.02 | 0.02 | 0.02 | 0.02 | 0.02 | 0.01 |
| Cumulative variance explained [%] | 99.79 | 99.82 | 99.84 | 99.87 | 99.89 | 99.91 | 99.93 | 99.95 | 99.97 | 99.99 | 100.00 |

Supplementary Table S2. Results of the MANCOVA on PC1–PC8 scores from the whole-sample PCA including TimePeriod, Sex, age-at-death, and log_10_ CS (*N* = 110).

|  | Pillai's Trace | *F* | *P* |
| --- | --- | --- | --- |
| TimePeriod | 0.875 | 85.75 | < 0.001 |
| Sex | 0.223 | 3.51 | 0.0013 |
| Age-at-death | 0.071 | 0.94 | 0.49 |
| log_10_CS | 0.176 | 2.62 | 0.012 |

Supplementary Table S3. Results of the ANOVA on log_10_ CS including TimePeriod and Sex (*N* = 110).

|  | *F* | *P* |
| --- | --- | --- |
| TimePeriod | 72.45 | < 0.001 |
| Sex | 85.92 | < 0.001 |
| TimePeriod×Sex | 1.98 | 0.16 |

Supplementary Table S4. Results of the MANOVA on PC1–PC8 scores from the whole-sample PCA including TimePeriod and Sex (*N* = 112).

|  | Pillai's Trace | *F* | *P* |
| --- | --- | --- | --- |
| TimePeriod | 0.881 | 93.18 | < 0.001 |
| Sex | 0.186 | 2.88 | 0.0063 |
| TimePeriod×Sex | 0.107 | 1.51 | 0.16 |

Supplementary Table S5. Results of the ANOVA on PC1–PC8 scores from the whole-sample PCA including TimePeriod and Sex (*N* = 112).

|  |  | PC1 | PC2 | PC3 | PC4 | PC5 | PC6 | PC7 | PC8 |
| --- | --- | --- | --- | --- | --- | --- | --- | --- | --- |
| TimePeriod | *F* | 0.0021 | 23.77 | 69.63 | 33.64 | 1.15 | 2.48 | 3.56 | 1.03 |
|  | *P* | 0.96 | < 0.001 | < 0.001 | < 0.001 | 0.29 | 0.12 | 0.062 | 0.31 |
| Sex | *F* | 12.40 | 0.42 | 0.16 | 2.91 | 5.46 | 0.08 | 0.0015 | 1.04 |
|  | *P* | < 0.001 | 0.52 | 0.69 | 0.091 | 0.021 | 0.78 | 0.97 | 0.31 |
| TimePeriod×Sex | *F* | 3.74 | 0.29 | 0.064 | 0.62 | 0.73 | 0.99 | 0.94 | 0.73 |
|  | *P* | 0.056 | 0.59 | 0.80 | 0.43 | 0.39 | 0.32 | 0.34 | 0.39 |

Supplementary Table S6. Results of the ANOVA on PC1–PC9 scores from the separate PCA for the historical samples including Sex (*N* = 56).

|  |  | PC1 | PC2 | PC3 | PC4 | PC5 | PC6 | PC7 | PC8 | PC9 |
| --- | --- | --- | --- | --- | --- | --- | --- | --- | --- | --- |
| Sex | *F* | 0.65 | 0.16 | 0.66 | 6.63 | 0.045 | 0.068 | 0.41 | 4.42 | 3.69 |
|  | *P* | 0.42 | 0.69 | 0.42 | 0.013 | 0.83 | 0.79 | 0.52 | 0.040 | 0.060 |

Supplementary Table S7. Results of the ANOVA on PC1–PC9 scores from the separate PCA for the present-day samples including Sex (*N* = 56).

|  |  | PC1 | PC2 | PC3 | PC4 | PC5 | PC6 | PC7 | PC8 | PC9 |
| --- | --- | --- | --- | --- | --- | --- | --- | --- | --- | --- |
| Sex | *F* | 16.82 | 0.61 | 3.64 | 0.52 | 0.064 | 0.019 | 5.26 | 7.94 | 2.50 |
|  | *P* | < 0.001 | 0.44 | 0.062 | 0.47 | 0.80 | 0.89 | 0.026 | 0.0067 | 0.12 |

Supplementary Table S8. Confusion matrix for the LDA with leave-one-out cross-validation.

|  |  |  | Predicted Sex | |  | Accuracy [%] |
| --- | --- | --- | --- | --- | --- | --- |
|  |  |  | F | M |  |  |
| True Sex | F |  | 30 | 19 |  | 61.2 |
|  | M |  | 18 | 45 |  | 71.4 |
|  |  |  |  | Overall |  | 67.0 |
|  |  |  |  | Balanced |  | 66.3 |

Supplementary Table S9. Results of the sensitivity analyses.

|  |  | PC1–PC5 | |  | PC1–PC8 | |  | PC1–PC10 | |  | PC1–PC12 | |  |
| --- | --- | --- | --- | --- | --- | --- | --- | --- | --- | --- | --- | --- | --- |
|  |  | *F* | *P* |  | *F* | *P* |  | *F* | *P* |  | *F* | *P* |  |
| TimePeriod |  | 93.55 | < 0.001 |  | 93.18 | < 0.001 |  | 78.30 | < 0.001 |  | 71.07 | < 0.001 |  |
| Sex |  | 4.44 | 0.0010 |  | 2.88 | 0.0063 |  | 3.17 | 0.0015 |  | 3.20 | < 0.001 |  |
| TimePeriod×Sex |  | 1.71 | 0.14 |  | 1.51 | 0.16 |  | 2.05 | 0.036 |  | 1.89 | 0.044 |  |
